# The Toll for Trafficking: Toll-Like Receptor 7 Delivery to the Endosome

**DOI:** 10.3389/fimmu.2017.01075

**Published:** 2017-09-04

**Authors:** Carlene Petes, Natalya Odoardi, Katrina Gee

**Affiliations:** ^1^Department of Biomedical and Molecular Sciences, Queen’s University, Kingston, ON, Canada

**Keywords:** toll-like receptor 7, single-stranded ribonucleic acid, endosomal trafficking, uncoordinated 93 homolog B1 (*Caenorhabditis elegans*), furin peptidases, asparagine endopeptidases

## Abstract

Toll-like receptor (TLR)-7 is an endosomal innate immune sensor capable of detecting single-stranded ribonucleic acid. TLR7-mediated induction of type I interferon and other inflammatory cytokine production is important in antiviral immune responses. Furthermore, altered TLR7 expression levels are implicated in various autoimmune disorders, indicating a key role for this receptor in modulating inflammation. This review is focused on the regulation of TLR7 expression and localization compared to that of the other endosomal TLRs: TLR3, 8, and 9. Endosomal TLR localization is a tightly controlled and intricate process with some shared components among various TLRs. However, TLR-specific mechanisms must also be in place in order to regulate the induction of pathogen- and cell-specific responses. It is known that TLR7 is shuttled from the endoplasmic reticulum to the endosome via vesicles from the Golgi. Several chaperone proteins are required for this process, most notably uncoordinated 93 homolog B1 (*Caenorhabditis elegans*), recently identified to also be involved in the localization of the other endosomal TLRs. Acidification of the endosome and proteolytic cleavage of TLR7 are essential for TLR7 signaling in response to ligand binding. Cleavage of TLR7 has been demonstrated to be accomplished by furin peptidases in addition to cathepsins and asparagine endopeptidases. Moreover, triggering receptor expressed on myeloid cells like 4, a protein associated with antigen presentation and apoptosis in immune cells, has been implicated in the amplification of TLR7 signaling. Understanding these and other molecular mechanisms controlling TLR7 expression and trafficking will give insight into the specific control of TLR7 activity compared to the other endosomal TLRs.

## Introduction

Toll-like receptors (TLRs) are a class of pattern recognition receptors that recognize bacterial or viral pathogen-associated molecular patterns (PAMPs), playing a key role in innate immune responses (1, 2). This family includes plasma membrane receptors identified in mice and humans: TLR1, TLR2, TLR4, TLR5, TLR6, and TLR10 (2, 3), nucleic acid-sensing endosomal receptors also identified in mice and humans: TLR3, TLR7, TLR8, and TLR9, and murine-specific endosomal receptors: TLR11, TLR12, and TLR13 (4–7). The TLR family members have been well reviewed as mediators of innate immunity, as well as being extensively linked to autoimmunity (8–14).

**Figure 1:**
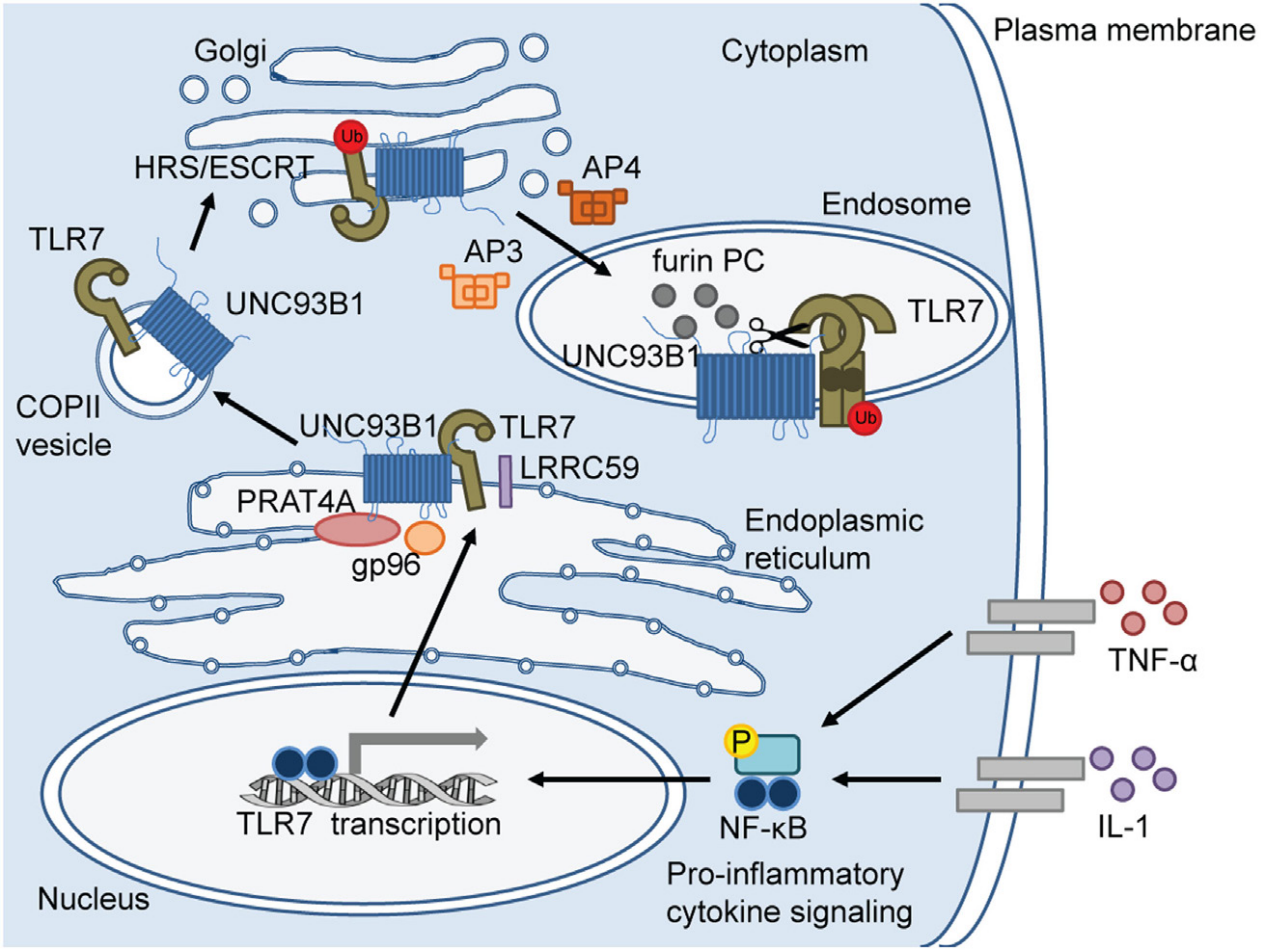
Toll-like receptor (TLR)-7 trafficking from the endoplasmic reticulum (ER) to the endosome. In response to pro-inflammatory cytokine signaling, TLR7 transcription is induced via nuclear factor (NF)-κB. TLR7 protein is synthesized in the ER where ER-resident chaperone proteins, gp96 and protein-associated with TLR4 (PRAT4A), are required to facilitate proper folding of TLR7 before exiting the ER. Upon cellular stimulation (e.g. imiquimod) or infection, leucine-rich repeat (LRR) containing protein 59 (LRRC59) promotes uncoordinated 93 homolog B1 (*Caenorhabditis elegans*) (UNC93B1)-mediated packaging of TLR7 into coat protein complex II (COPII)-coated vesicles to exit the ER and translocate to the Golgi. In the Golgi, UNC93B1 ubiquitinates TLR7 and HRS/ESCRT machinery sorts ubiquitinated TLR7 for endolysosomal transport. Adaptor protein (AP)-4 and AP-3 deliver TLR7 from the Golgi to lysosome-related organelles, such as “NF-κB endosomes” and “IRF-7 endosomes,” respectively. Once TLR7 reaches the endosome, endosomal acidification occurs for proteolytic cleavage by proteases, including furin proprotein convertases (PC). TLR7 cleavage separates the N-terminal ectodomain (darker circle) from the C-terminal ectodomain, transmembrane domain, and cytosolic region. The N terminal region forms disulfide bonds with the C-terminal region and remains in the endosome for optimal signaling.

Of the endosomal TLRs, TLR3 recognizes dsRNA and TLR9 recognizes dsDNA. Alternatively, TLR7 and the closely related TLR8 respond to purine-rich single-stranded ribonucleic acid (ssRNA) to elicit an immune response to pathogens which are recognized in the endosome (15–17). Thus, localization of these TLRs to the endosome is a requirement for detection of viral and bacterial nucleic acids that are endocytosed. Control of the trafficking pathways of these TLRs to the endosome is a regimented process involving several steps. In the literature, there is a greater emphasis on the regulation of TLR9 localization, while knowledge of the mechanisms controlling TLR7 localization is mostly deduced from TLR9 studies (18–20). The steps involved in endosomal localization of TLR7 are outlined in Figure 1 and are discussed in detail in this review.

TLR7 and TLR8 are often referred to together in the literature due to their high degree of homology and similarity in function. However, in comparison to TLR7, the mechanisms controlling TLR8 localization are less understood, likely due to a lack of appropriate murine model systems for studying TLR8. For example, the commonly used synthetic RNA analog resiquimod (R848) triggers human TLR7 and TLR8 signaling and murine TLR7 signaling, but not murine TLR8 (15, 21). Moreover, many of the studies delineating the mechanisms behind TLR7 localization have been performed in murine models and few studies have focused solely on human cells. Therefore, throughout this review, the studies discussed were performed in murine models unless otherwise specified. As well, much of the published work has examined TLR7 responsiveness, leaving a void in the literature regarding TLR8-specific mechanisms. Herein, we provide an in-depth commentary on the regulation of TLR7 expression and function and include additional highlights on mechanisms which control TLR8 localization.

### Expression Patterns of TLR7

The distribution of TLR7 expression among various cell types is differentially regulated. Constitutive expression of TLR7 is predominant in human and murine plasmacytoid dendritic cells (pDCs) and B cells compared to other circulating immune cells (22–24). Low levels of TLR7 have also been observed in non-immune cells such as hepatocytes, epithelial cells, and keratinocytes (25–28). Unlike TLR7, TLR8 is more strongly expressed in myeloid cells and to a lesser degree in pDCs (22, 29). Under certain circumstances, TLR7 expression is inducible in cells expressing low to undetectable basal TLR7 levels, including immune cells such as macrophages and myeloid DCs, and non-immune cells such as hepatocytes and keratinocytes (25, 28, 30–32). For example, interferon (IFN)-γ stimulation of human macrophages resulted in the induction of TLR7 mRNA expression and consequently enhanced TLR7 responsiveness (16). As well, infection with viruses such as hepatitis C virus (HCV), human immunodeficiency virus (HIV), and influenza A virus (IAV) also result in upregulation of TLR7 expression in hepatocytes, circulating immune cells, and primary macrophages, respectively (25, 30, 33). Under these circumstances, it is likely that the cytokines induced by virus infection are responsible for triggering the induction of TLR7 expression. Indeed, influenza virus infection of human primary macrophages results in upregulation of TLR7 mRNA in a type I IFN-dependent manner (33). Furthermore, augmented TLR7 expression is observed in cancer and autoimmune diseases such as rheumatoid arthritis and systemic lupus erythematosus (SLE) (31, 34, 35). Other cytokines and PAMPs released during inflammation as a result of infection or disease likely play a role in the regulation of constitutive and inducible TLR7 expression.

Given that the expression levels of TLR7 are relatively well documented under both normal and pathogenic conditions, it is surprising that the molecular mechanisms involved in the signaling pathways controlling TLR7 induction during virus infection or autoimmune conditions have not been well-described. As well, the regulation of constitutive TLR7 expression is relatively understudied. In a study by Lee et al., the TLR7 putative promoter sequence was cloned and characterized (36). Through computer analysis of the human *TLR7* gene sequence (accession number: NT_011757), binding sites for the following transcription factors were identified: nuclear factor (NF)-κB, C/EBPα, C/EBPβ, GR, IFN regulatory factor (IRF)-1, MIG1, NF-1, Oct-1, RAP1, RSRFC4, RxR-β, SRF, SP1, and SRY (36). Using a human liver cell line as a model system, TLR7 expression induced by tumor necrosis factor (TNF)-α and interleukin (IL)-1 treatment was shown to require NF-κB activation but not that of IRF-7 (36). Further investigation of the signaling pathways regulating TLR7 expression is warranted in both immune and non-immune cells, particularly in inflammatory conditions.

### Antiviral Functions of TLR7

Immune responses to ssRNA viruses include production of TLR7-mediated type I IFNs, a family of key antiviral cytokines that induce a variety of genes collectively known as IFN-inducible genes. Viruses have adapted a number of evasive strategies to avoid and/or shut down type I IFN responses, and there are a variety of innate viral sensors responsible for induction of type I IFN; these include melanoma differentiation-associated protein 5 (MDA5), laboratory of genetic and physiology 2 (LGP2), mitochondrial antiviral-signaling protein (MAVS), stimulator of IFN genes (STING), and retinoic acid inducible gene (RIG)-I (37). The role of these molecules during virus infection has been reviewed in detail elsewhere (37, 38). It is interesting to note that these sensors often work in tandem with each other and/or TLR7 to respond to infection. For example, it has been shown that TLR7 ligation induces expression of the cytoplasmic viral RNA-sensing helicase, RIG-I, in order to clear virus infection, thereby linking TLR7 and RIG-I function (39). Specifically, pDCs generally express low RIG-I but upon stimulation with the TLR7 agonist imiquimod, RIG-I is upregulated and thus this allows for cooperative RIG-I- and TLR7-mediated viral clearance (39).

TLR7 is well-established to bind viral ssRNA; specific examples include vesicular stomatitis virus (VSV), IAV, HIV-1, hepatitis B virus, and HCV (25, 40–43) or synthetic guanine-rich RNA sequence analogs such as R848, gardiquimod, loxoribine, and most notably the human papillomavirus treatment, imiquimod (15, 44, 45). Upon virus infection or agonist stimulation, ssRNA enters the endosome via autophagy or receptor-mediated endocytosis, as discussed in detail further below (46, 47). Dimeric TLR7 then interacts with the ssRNA which subsequently initiates signal transduction (48, 49). Signal transduction of TLR1-10 has been thoroughly reviewed in the literature (1–3, 8, 50–53). Similar to all TLRs, TLR7 contains a Toll/IL-1 receptor domain that associates with myeloid differentiation primary response gene 88 (MyD88) for signal transduction, aside from TLR3 which signals via the MyD88-independent pathway (20, 54). Unlike TLR8 and TLR9, which exist as preformed dimers (55–57), TLR7 dimerizes upon ligand binding in the endosome to initiate TLR7-mediated MyD88 signal transduction (58). This results in the subsequent activation of mitogen-activated protein kinase (MAPK) cascades, NF-κB activation (53, 59), as well as IRF-7 (60, 61) and IRF-5 (62, 63) activation via IL-1 receptor-associated kinases (IRAK)-1/2/4 and TNF receptor-associated factor-3/6 (61, 64, 65). Signaling in human immune cells by TLR7 has been documented to trigger production of pro-inflammatory cytokines including TNF-α, IL-6, IL-1β, IL-12, and IFN-α (61, 66–69). Although the specific signaling pathways triggered by TLR7 activation have been described (53, 60, 61), the underlying mechanisms controlling TLR7 trafficking to the endosomal compartment are now coming into the limelight.

## TLR7 Endosomal Localization

Like the other endosomal TLRs, TLR7 can only bind and respond to its ligand in the endosome to prevent recognition of self-genomic information (70). TLR7 is directed to the endosome by an intricate shuttling process mediated by several chaperone proteins as illustrated in Figure 1. Furthermore, proteolytic cleavage must occur for ligand recognition (71, 72). Details regarding TLR7 localization compared to that of the other endosomal TLRs are discussed below.

### Exit of TLR7 from the Endoplasmic Reticulum (ER)

TLR7 is initially produced as an inactive protein in the ER and must be correctly folded prior to exiting the ER. Two ER-resident chaperone proteins, the heat shock protein gp96 and protein-associated with TLR4 (PRAT4A), are involved in the folding of TLR7 as well as other TLRs, with the exception of TLR3 (Figure 1) (73–75). In the absence of functional gp96, macrophages, and B cells are unable to respond to TLR7 ligation (73, 74). As well, in PRAT4A-deficient macrophages, TLR7 responsiveness was completely impaired (75). A different ER-resident chaperone protein, leucine-rich repeat (LRR) containing protein 59 (LRRC59), was demonstrated to mediate TLR8 localization as well as TLR3 and TLR9 in human cells (76). Other experiments from the same study also imply that TLR8 and TLR9 endosomal localization requires LRRC59, as the absence of this protein resulted in decreased TLR8 and TLR9 signaling; however, TLR8 and TLR9 localization was not directly measured (76). LRRC59 could also be required for TLR7 signaling, however, such studies are necessary for confirmation. On the other hand, it is interesting to speculate that regulation of TLR7 localization diverges from the other TLRs and evidence to support this is outlined in the sections below.

One of the more researched ER-resident chaperone proteins, uncoordinated 93 homolog B1 (*Caenorhabditis elegans*) (UNC93B1), has a clearly defined role in the export of nucleotide-sensing TLRs from the ER to the Golgi and endosomes (77–81). UNC93B1 interacts with the TLRs to mediate packaging into coat protein complex II (COPII)-coated vesicles. The COPII complex is composed of conserved coat proteins and is involved in promoting anterograde protein transport from the ER to the Golgi; thus TLR-containing COPII vesicles bud from the ER for transport to the Golgi (19, 79). UNC93B1 is thought to be involved in ubiquitination of TLR9 and potentially TLR7 in a post-ER compartment to facilitate transfer and interactions with other chaperone proteins (82). Once in the Golgi, HRS/endosomal sorting complex required for transport (HRS/ESCRT) machinery sorts ubiquitinated TLR7 for endolysosomal transport through the non-canonical ESCRT pathway to maintain receptor stability within the endosome (Figure 1) (70, 82).

In murine models, different mutations of the UNC93B1 gene, *Unc93b1*, highlight the role of this protein in TLR shuttling. The UNC93B1 H412R mutation, also called the 3D (or triple D) mutation, causes inefficient processing of TLR3, TLR7, and TLR9 and results in enhanced susceptibility to virus infection (19, 77–79, 81). Similarly, the “loss of endosomal TLR response” (*Letr*) mutation, a 54-amino acid deletion in exon 4 of murine *Unc93b1*, leads to increased viral load of IAV (83), likely due to compromised TLR7 trafficking out of the ER. Interestingly, in pDCs, macrophages, B cells, and conventional DCs, the D34A mutation of UNC93B1 leads to increased cytokine production upon TLR7 activation, while cytokine production upon TLR9 activation was decreased and that of TLR3 was unaffected (84, 85). Taken together, these studies suggest that modulation of TLR-UNC93B1 binding capacity may fine-tune specific endosomal TLR responses.

UNC93B1 mRNA expression and protein levels are upregulated upon IFN-α, -β, or -γ stimulation of murine macrophages (86), indicating that antiviral IFN production may, in turn, enhance endosomal TLR7 localization and downstream signaling. However, the direct effect of type I IFN signaling on TLR7 localization has not been well documented. Furthermore, while in the ER, TLR7 competes with TLR9 for interaction with UNC93B1, and TLR9 generally prevails (84, 85, 87). Thus, IFN-mediated upregulation of UNC93B1 represents a potential mechanism to enhance TLR7 trafficking to endosome by increasing the likelihood of TLR7-UNC93B1 interaction over that of TLR9. Additionally, stimulation with imiquimod, as well as with TLR9 or TLR4 agonists, triggers UNC93B1-dependent TLR7 endosomal localization in murine myeloid cells (79). Furthermore, modeling in the human embryonic kidney (HEK)-293 cell line demonstrated that stimulation via other TLRs, in particular that of TLR8, as well as non-specific induction of endocytosis, enhances LRRC59 binding to UNC93B1 (76), indicating that endocytic events, such as virus invasion, may augment endosomal TLR localization to enhance the antiviral response.

### Packaging TLR7 in the Endosome

Compared to other chaperone proteins, UNC93B1 appears to be unique in that it also leaves the ER to accompany the TLR in transit to the endosome. Other proteins are required for TLR-UNC93B1 transit to the endosome, these include the adaptor protein (AP) family (AP-1–4), which function to sort membrane proteins (88). Interactions between the APs, UNC93B1, and TLRs appear to be complex, with evidence for direct interaction between APs and UNC93B1 (19, 81) and APs with TLRs (19). Differential requirements for AP-1, AP-2, AP-3, and AP-4 have been proposed for the individual endosomal TLRs. Specifically, AP-4 binds TLR7 to sort into COPII vesicles for transport to the Golgi in macrophages (Figure 1) (19). TLR7 directly translocates from the Golgi to the endosome, accompanied by both UNC93B1 and AP-4 (19). Unlike TLR7, TLR9 is first shuttled from the Golgi to the plasma membrane, then to the endosome in an AP-2-dependent manner (19). Type I IFN induction in response to TLR7 ligation was found to depend on AP-3 for shuttling to specialized lysosome-related organelles (LRO) (89). These LRO, called “IRF-7 endosomes,” are lysosome-associated membrane protein (LAMP)-1^+^/LAMP2^+^ and signal via IRF7 to produce type I IFN (89). In contrast, pro-inflammatory cytokine production occurs upon TLR ligation in vesicle-associated membrane protein-3^+^/LAMP2^−^ LRO that signal by NF-κB, called “NF-κB endosomes” (89). AP-3 deficiency abrogated TLR7-mediated type I IFN production in pDCs, while NF-κB-induced cytokine production was maintained, indicating a specific role for AP-3 in shuttling TLR7 to IRF-7 endosomes (89). Interestingly, Lee et al. demonstrated a direct interaction between UNC93B1 and AP-2, but not AP-1, 3, or 4, as well as a direct interaction between TLR7 and AP-4, but not AP-1, 2, or 3 (19). This suggests that other sorting proteins may be required to facilitate TLR7 travel to IRF-7 endosomes in tandem with AP-3. Furthermore, requirements for the targeting of TLR7 to NF-κB endosomes have not been elucidated and thus represent a focus for future research.

## TLR7 Cleavage

Restriction of nucleic acid-sensing TLRs to the endosome, as well as the dependence on cleavage for activation both help prevent recognition of self-DNA/RNA (90, 91). Like all endosomal TLRs, prior to gaining the capacity to bind and respond to ligands, TLR7 must be proteolytically cleaved (92). In general, endosomal TLRs are cleaved by a common mechanism initiated by decreased endosomal pH, required for enzymatic activity. The cleavage events are a complex process and are thought to involve a combination of asparagine endopeptidase (AEP) and/or multiple cathepsins (71, 72, 90, 92). The majority of studies focusing on the regulation of cleavage events used a TLR9-expressing murine macrophage cell line (RAW cells) as a model system. Indeed, the initial role for cathepsins in TLR9 was suggested by Ewald et al.; however, at the time, this group was unable to experimentally block cathepsin activation efficiently, and they concluded that a combination of proteases were likely responsible (90). Later, the same group demonstrated that in RAW cells and murine conventional DCs, TLR cleavage was a two-step event. The initial cleavage event could be performed by either AEP or cathepsin, indicating a redundant role for these proteins (72). The second cleavage step for optimal signaling responses is referred to as TLR trimming and appears to rely exclusively on cathepsin activity (72). In the same report, TLR7 cleavage was shown to require both AEP and cathepsin activity (72). The need for AEP-dependent TLR7 cleavage was further highlighted by inefficient TLR7 signaling in DCs, which was linked to poor induction of adaptive immune responses during IAV infection of AEP-deficient mice (92).

Interestingly, Hipp et al. found that the furin-like proprotein convertases (PC), part of the family of serine endoproteases including furin, PC5/6, and PC7, cleave human TLR7 in the endosome for the generation of competent TLR7 signaling responses (Figure 1) (93). This study demonstrated a novel mechanism for cleavage of human TLR7 compared to the numerous studies focused on murine endosomal TLR7 cleavage by cathepsins and AEP (71, 72, 90, 92). Likewise, furin-like proteases have also been implicated in TLR8 proteolysis in primary human monocytes and macrophages (94). Thus, the use of furin-like proteases may be specific to TLR7 and TLR8 and may represent an alternative mechanism regulating TLR7 cleavage, in addition to AEP and cathepsins. The dependence for TLR7 cleavage on AEP, cathepsins, or furins could be dictated by cell type and differences in human and murine cell models.

With regards to the specific amino acid sequences for cleavage sites, TLR7 is susceptible to proteolysis between two LRR domains (LRR14–15) which correspond to residues 450–479 containing asparagine 478, required for AEP-mediated cleavage (92). The role of cleavage in TLR7 localization versus signaling was demonstrated using a model of murine fibroblasts and bone marrow-derived DCs (BMDC) transfected with wild type or mutated TLR7. Mutation of asparagine 478 to glutamine (N478Q) did not hinder TLR7 localization; however, downstream signaling in response to imiquimod was inhibited (92). This cleavage site separates the N-terminal (ectodomain) from the C-terminal (ectodomain, transmembrane, and cytosolic regions) that remains in the endosome for downstream signaling; it is suggested that the TLR7 N-terminus is cleaved but linked to the C-terminus by disulfide bonds and is required for endosomal localization and ligand binding in both murine DCs and human cell lines (THP-1; HEK-293T) (Figure 1) (95, 96). Crystallography studies of human TLR8 and studies in primary human myeloid cells also demonstrate N- and C-terminal association after cleavage (57, 94). Taken together, this information combined with that on the enzymatic cleavage highlights the complexity of how processing of TLR7, as well as TLR8, is regulated.

### Ligand Delivery to TLR7

Like the regulation of TLR7 trafficking, ligand delivery to TLR7 is also an intricately regulated process. Upon infection, agonist stimulation, or damage/apoptosis to neighboring cells, ligands must enter the endosome to trigger TLR7 activation. Receptor-mediated endocytosis can deliver virus or purified TLR7 ligands to endosomal TLR7 (48, 49, 97–99). In autoimmunity, delivery of self-ligand to the endosome relies on proteins such as high mobility group box 1 protein and the cathelicidin, LL37 (100). Thus, TLR7 responses are regulated during inflammatory disease conditions by release or overexpression of these molecules; for example, increased LL37 expression is associated with exacerbated TLR7-mediated cytokine production during autoimmune disease (101). As well, B cells bearing B cell receptors (BCR) specific for DNA, and pDCs bearing the Fc receptor FcγRIIa (CD32), were shown to internalize self-DNA for delivery to TLR9 in SLE (102, 103). This sequential engagement of BCR and TLR9 was recapitulated for TLR7 where autoreactive BCRs were shown to bind and internalize ssRNA for delivery to TLR7 in a model of SLE (104).

While viral ssRNA may reach the endosome as a result of endocytosis, murine pDCs have also been shown to utilize autophagy, a homeostatic process whereby damaged or unnecessary cellular material from the cytoplasm is degraded in lysosomes, to serve an endosomal delivery platform for viral RNA-TLR7 ligation (46, 105, 106). Autophagosomes are formed by a sequential series of steps, each involving autophagy-related gene (ATG) proteins; the process is initiated by phagophore formation, a double-membrane vacuole that expands into an autophagosome (107). Once the autophagosome fuses with the endosome or lysosome, autolysosomes are formed for degradation of cellular material (108). A role for autophagy and TLR7 ligand delivery during viral infection is prominent in murine pDCs since these cells exhibit TLR7-dependent type I IFN production in response to virus infection (47). Lee et al. demonstrated that murine ATG5-deficient pDCs infected with VSV were unable to produce IFN-α (47). A few years later, two other groups demonstrated that autophagy was required for TLR7-mediated IFN-α production in primary human pDCs infected with HIV-1 or paramyxovirus simian virus 5 (109, 110). Of note, autophagy has also been implicated in ssRNA delivery to TLR7 in murine B cells in SLE (111). Preference between endocytosis and autophagy for ligand delivery during virus infection may depend on several factors, such as the mode of breaching the cell (endocytosis vs membrane fusion), replication strategies, and expression of viral inhibitory factors. In addition to the role for autophagosome-mediated delivery of TLR7 ligands to the endosome, a role for TLR7 signaling in the induction of autophagy has also been investigated (46). Upon examination of TLRs 1 through 9, ligation of TLR7 demonstrated the most potent induction of autophagy in murine macrophages (46). Furthermore, similar to TLR4, TLR7 induces Beclin 1 (mammalian homolog of yeast ATG6) interaction with MyD88 for enhanced autophagosome formation in murine macrophages (46, 112, 113). These studies provide evidence for a potential positive feedback mechanism between TLR7 signaling and autophagy-mediated viral recognition.

### Modulation of TLR7 Signaling

Investigations into the roles for the triggering receptor expressed on myeloid cells (TREM) receptor family and associated ligands have identified these proteins as novel constituents involved in modulating TLR signaling and responsiveness (114–117). Recently, TREM like 4 (TREML4) has been characterized to amplify TLR7 signaling in murine and human cells (Figure 1) (118). Little is known regarding TREML4 functions; it is predominantly found on splenic macrophages as well as CD8^+^ DCs, and it has been shown to bind apoptotic cells and enhance antigen presentation to T cells (119, 120). In *Treml4*-deficient BMDC, decreased TLR7 endosomal localization and TLR7-MyD88 interaction was observed with a concomitant decrease in activation of TLR7-mediated signaling and cytokine production (118). Knockdown of *Treml4* in human macrophages also resulted in decreased cytokine induction in response to gardiquimod (118). Together, this indicates that upon TLR7 ligation TREML4 may function to stabilize the TLR7-MyD88 interaction, thus promoting signal transduction and resulting cytokine production. Interestingly, a role for TREML4 was also suggested for TLR9 and TLR13 signaling (118), however, the direct impact of TREML4 deficiency on TLR9 or TLR13 association with MyD88 or endosomal localization was not investigated. The cellular localization patterns of TREML4 have not been identified and whether TREML4 is recruited to the endosomes with TLR7 or if it directly interacts with TLR7 remains unknown. Interestingly, stimulation of splenic macrophages with gardiquimod or CpG DNA, a TLR9 agonist, resulted in enhancement of TREML4 mRNA expression (118), although stimulation of splenic DCs with TLR3, TLR4, or TLR9 agonists did not impact TREML4 expression in another study (119). This suggests that TREML4 may play a role in a positive feedback loop for TLR7 signaling and may also have differential effects depending on the cell type. These studies signify a novel mechanism for TLR7 signal amplification in the endosome and this represents an enticing new avenue for investigation to delineate signaling requirements for endosomal TLRs.

Another mechanism to modulate TLR signaling occurs upon multiple subsequent exposures to ligand, which results in abrogation of TLR responsiveness. This is best characterized with TLR4 stimulation using lipopolysaccharide (LPS), whereby LPS (endotoxin) tolerance is characterized by reduced TNF-α and IL-6 production upon repeated LPS stimulation in comparison to a single exposure to LPS (121). Interestingly, TLR7 tolerance has been observed in murine macrophages repeatedly exposed to TLR7 agonists; repeated exposure to imiquimod induced a refractory state whereby cells did not respond to subsequent TLR7 stimulation, due to elevated MyD88 pathway inhibitors: IRAK-M and Src homology 2 domain-containing inositol-5-phosphatase-1, and impaired activation of NF-κB, MAPK p38, and c-Jun N-terminal kinase (122, 123). TLR “hetero-tolerance,” accomplished by exposure to one TLR agonist resulting in decreased responsiveness to secondary stimulation by an agonist for a different TLR, also serves as a general mechanism to control TLR responses. For example, *in vivo* intravenous administration of LPS in human subjects followed by *ex vivo* leukocyte challenge with TLR7 ligand (S-27609) led to suppressed secretion of TNF-α, IL-6, IL-1β, and IL-10 compared to S-27609 stimulation alone (124). Moreover, TLR7 ligand stimulation reduces subsequent responses to other TLR agonists. For example, human and murine myeloid cells cultured with R848 or loxoribine exhibited decreased TLR4-responsiveness (125–128). Furthermore, pretreatment of human macrophages with R848 inhibits subsequent induction of TLR2, TLR4, TLR5, and TLR7 signaling (125). Interestingly, costimulation of murine macrophages with the TLR7 agonist, gardiquimod, and the TLR9 agonist, CpG DNA, resulted in a suppressor of cytokine signaling-1-mediated reduction of TNF-α and IL-6 (129). These studies demonstrate multiple mechanisms that regulate TLR responsiveness and further indicate that TLR7 activity can modulate responses to other TLR molecules and vice versa.

## Future Perspectives

Elucidating the mechanisms controlling TLR7 localization is critical to further our understanding of key elements that dictate TLR7 ligand binding and downstream signaling. TLR7 and TLR8 appear to be redundant, and although there is a paucity of information on the regulation of TLR8 localization, subtle differences do exist between TLR7 and TLR8 regarding the regulation of endosomal localization and function. Thus, understanding precisely how TLR8 is differentially regulated from TLR7 is a key area for future research. Moreover, gaining a better understanding of the differential requirements for localization of each of the different endosomal TLRs will facilitate identification of mechanisms controlling ligand delivery. Ultimately, in-depth analysis of the complex processes regulating the localization and function of TLR7 will garner further information on fine-tuning immune responses in virus infection, cancer, and autoimmune diseases for the development of novel therapeutics.

## Author Contributions

CP, NO, and KG contributed to the conceptualization, discussion, and writing of this review. CP designed and composed the figure.

## Conflict of Interest Statement

The authors declare that the research was conducted in the absence of any commercial or financial relationships that could be construed as a potential conflict of interest.
